# Three-Month Real-Time Dengue Forecast Models: An Early Warning System for Outbreak Alerts and Policy Decision Support in Singapore

**DOI:** 10.1289/ehp.1509981

**Published:** 2015-12-11

**Authors:** Yuan Shi, Xu Liu, Suet-Yheng Kok, Jayanthi Rajarethinam, Shaohong Liang, Grace Yap, Chee-Seng Chong, Kim-Sung Lee, Sharon S.Y. Tan, Christopher Kuan Yew Chin, Andrew Lo, Waiming Kong, Lee Ching Ng, Alex R. Cook

**Affiliations:** 1 Environmental Health Institute,; 2 Environmental Public Health Operations Department, and; 3 Centre for Climate Research Singapore, National Environment Agency, Singapore; 4 School of Engineering, Nanyang Polytechnic, Singapore; 5 School of Biological Sciences, Nanyang Technological University, Singapore; 6 Saw Swee Hock School of Public Health, National University of Singapore and National University Health System, Singapore; 7 Yale-NUS College, National University of Singapore, Singapore

## Abstract

**Background:**

With its tropical rainforest climate, rapid urbanization, and changing demography and ecology, Singapore experiences endemic dengue; the last large outbreak in 2013 culminated in 22,170 cases. In the absence of a vaccine on the market, vector control is the key approach for prevention.

**Objectives:**

We sought to forecast the evolution of dengue epidemics in Singapore to provide early warning of outbreaks and to facilitate the public health response to moderate an impending outbreak.

**Methods:**

We developed a set of statistical models using least absolute shrinkage and selection operator (LASSO) methods to forecast the weekly incidence of dengue notifications over a 3-month time horizon. This forecasting tool used a variety of data streams and was updated weekly, including recent case data, meteorological data, vector surveillance data, and population-based national statistics. The forecasting methodology was compared with alternative approaches that have been proposed to model dengue case data (seasonal autoregressive integrated moving average and step-down linear regression) by fielding them on the 2013 dengue epidemic, the largest on record in Singapore.

**Results:**

Operationally useful forecasts were obtained at a 3-month lag using the LASSO-derived models. Based on the mean average percentage error, the LASSO approach provided more accurate forecasts than the other methods we assessed. We demonstrate its utility in Singapore’s dengue control program by providing a forecast of the 2013 outbreak for advance preparation of outbreak response.

**Conclusions:**

Statistical models built using machine learning methods such as LASSO have the potential to markedly improve forecasting techniques for recurrent infectious disease outbreaks such as dengue.

**Citation:**

Shi Y, Liu X, Kok SY, Rajarethinam J, Liang S, Yap G, Chong CS, Lee KS, Tan SS, Chin CK, Lo A, Kong W, Ng LC, Cook AR. 2016. Three-month real-time dengue forecast models: an early warning system for outbreak alerts and policy decision support in Singapore. Environ Health Perspect 124:1369–1375; http://dx.doi.org/10.1289/ehp.1509981

## Introduction

Dengue is an acute infectious disease common to tropical and subtropical regions. Dengue viruses are transmitted by *Aedes* mosquitoes, mainly *Aedes aegypti* and *Aedes albopictus* (Rosen et al. 1983). Globally, the World Health Organization has estimated that there are 50–100 million dengue infections per year (Rigau-Pérez et al. 1998), although more recent estimates have elevated this figure to 390 million, of which ~96 million are symptomatic (Bhatt et al. 2013). Dengue infection in humans is mostly self-limiting—although antiviral drugs are under development (Lim et al. 2013; Rathore et al. 2011)—but may require hospital admission, and the more severe manifestations of dengue may lead to death (Murphy and Whitehead 2011). Case fatality rates of dengue fever and severe dengue vary from 0–5% to 3–5% (Halstead 1999).

The city-state of Singapore, which lies approximately 130 km north of the equator, has a tropical rainforest climate in the Köppen–Geiger climate classification system (Peel et al. 2007) with no distinctive seasons. The climate, combined with Singapore’s highly urbanized environment, favors the presence of *Aedes* mosquitoes and the transmission of dengue virus (Thu et al. 1998), thus making Singapore highly vulnerable to dengue outbreaks. All four serotypes are endemic to Singapore, and there is frequent introduction and circulation of different genotypes of the virus (Lee et al. 2010, 2012). With an annual reported incidence in the range of 20–330 per 100,000 people, the economic impact of dengue in Singapore from 2000 to 2010 was estimated to be 850 million USD–1.15 billion USD, or approximately 200 USD per capita per year (Carrasco et al. 2011). Since 2003, > 100 dengue-related deaths have been reported [Ministry of Health, Singapore (MOH) 2012].

Antiviral drugs and vaccines have yet to reach the market (Douglas et al. 2013), and initial results from trials have been discouraging (Halstead 2012; Mahalingam et al. 2013; Sabchareon et al. 2012). In the absence of an effective vaccine against dengue, suppressing the mosquito vector population remains the key thrust of Singapore’s dengue-control program (Lee et al. 2013). From 2000 to 2009, the country spent > US$85 million per annum (Carrasco et al. 2011) on this endeavor. Since 2006, the National Environment Agency (NEA) has introduced virological surveillance for early warning of outbreaks (Lee et al. 2010) and a novel mosquito-breeding index that estimates the spatial distribution of *Ae. aegypti*, the main dengue vector in Singapore. Previous predictive capability relied on a qualitative understanding based on temperature, circulating serotype, vector data from the field, and estimated immunity level of the human population. If automated, statistical models hold the promise of being able to provide real-time quantitative forecasts of the appearance and evolution of a dengue outbreak, which may be used to efficiently guide the deployment of vector-control operations.

Any statistical approach to forecast dengue would need to meet certain criteria to be practical: *a*) use only data that are available at the time the forecast is made; *b*) be capable of forecasting weeks or months into the future to give lead time for preparing a public health response (for instance, hiring new control staff); *c*) possess validated and demonstrated predictive performance using data that were not used in its construction, to prevent over-fitting and to ascertain confidence levels; and lastly, *d*) be able to process new data rapidly. Population dynamic modeling of dengue exploits epidemiological and entomological knowledge and is valuable for modeling what-if scenarios, such as the effect of introducing changes to the host or mosquito population [such as introducing vaccination into the pediatric vaccination schedule (Coudeville and Garnett 2012) or releasing genetically modified sterile mosquitoes or those infected by *Wolbachia* (Hughes and Britton 2013)], and may be useful for predicting long-term changes to epidemic dynamics caused by changing levels of herd immunity or by the age structure of a population (Cummings et al. 2009; Egger et al. 2008). A limitation of population dynamic models is that they are difficult to integrate with real-time data streams, such as meteorological or incidence data. Although success has been achieved for epidemiologically simpler diseases such as influenza (Baguelin et al. 2010; Ong et al. 2010), the complexity caused by the possibility of having several serotypes circulating simultaneously and by the influence of the environment on the vector makes these models a relatively unattractive choice for short-term forecasts of dengue. In contrast, correlative statistical approaches—which describe the phenomenon but not the underlying process—are well suited to integration with multiple live data streams and may have good predictive accuracy if future conditions do not stray too far from the conditions used to parameterize them.

Other researchers have sought to develop statistical time series models to predict dengue in Singapore. Earnest et al. (2012) compared 1-week-ahead dengue forecasts based on two popular modeling methods—the autoregressive integrated moving average (ARIMA) and the Knorr-Held two-component (K-H) model—and showed that the K-H model was slightly more accurate. Hii et al. (2012a, 2012b) developed a forecasting model using Poisson multivariate regression to predict the number of cases over a 4-month interval, demonstrating that past temperature and rainfall data are good predictors of future dengue incidence. Another study (Althouse et al. 2011) predicted 1-week and 1-month dengue incidence in Singapore and Bangkok, respectively, based on internet search engine queries that might signal the early stages of an outbreak. The authors compared three statistical approaches based on these data—step-down linear regression, generalized boosted regression, and negative binomial regression—and concluded that the step-down linear model was superior to the other two. Although the aforementioned models meet many of the criteria noted above, it is noteworthy that none has been validated against data not used in its construction, and none was developed explicitly for operational use, suggesting that their predictive performance and usefulness to operations were not tested.

In this paper, we describe a new approach to forecasting dengue that is used by Singapore’s NEA in planning vector control and in public communication. The model specifically optimizes predictive accuracy over a 3-month time horizon with model complexity selected, and predictive performance evaluated, using out-of-sample forecasting. We show that this approach, which uses the least absolute shrinkage and selection operator (LASSO) method to fit large regression models, has better predictive performance than other modeling approaches.

## Materials and Methods

### Statistical Analyses

***LASSO.*** The Least Absolute Shrinkage and Selection Operator (LASSO) is a technique that was proposed in the 1990s (Tibshirani 1996) and has inspired much interest in the statistical methodology community on “small *n* large *p*” problems (Tibshirani 1996). This framework extends standard regression and related models such as logistic regression by simultaneously selecting which parameters to include in the model and what their values should be. Rather than optimizing the (log) likelihood *L*(*y*|β, *x*) for dependent variable *y*, independent variables *x* and coefficients β, as in standard regression, LASSO optimizes the sum of the log-likelihood and a penalty term controlled by an additional parameter λ, which controls model complexity. In particular, the optimal coefficients are the βs that maximize


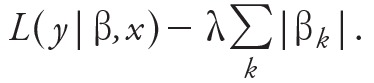
[1]

The penalty term λ controls model complexity: for a specific value of the penalty parameter, the optimal fit will have some (or many) coefficients set to 0, that is, some covariates are not used in the model. The penalty term is usually selected by cross-validation, in which *a*) the data are partitioned into several training and test sets; *b*) for each training set, a series of values of λ are considered; and *c*) for each λ, the best values of β are found and then used to predict the test data so that the out-of-sample performance can be measured. The value of λ that maximizes the average out-of-sample performance is then used to select the final model, implicitly meaning that the model complexity is selected to optimize predictive accuracy for a given set of independent variables. We used the glmnet algorithm (Friedman et al. 2010) implemented in the R statistical language (R Core Team 2014) to fit the models.

Our approach was to develop a tailored submodel unique to each forecast window from 1 week to 12 weeks in the future. For each submodel, the outcome variable was the weekly number of cases (natural log–transformed, with 1 added to avoid logging 0), and a large set of potential input variables were considered (details may be found in the data section). The formulation for each submodel was a multivariable linear regression. LASSO was used to select a (potentially different) set of predictors for each forecast window along with the values of their coefficients, with 10-fold cross-validation used to determine optimal model complexity, before the forecasts were “stitched together” graphically to create the impression of a single predictive routine.

**Figure 1:**
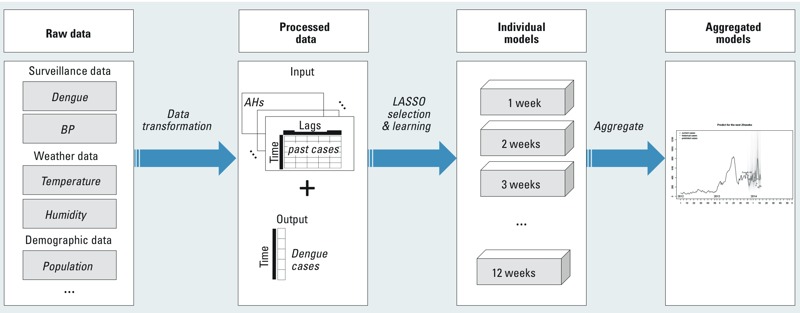
Model framework. Raw data from 2001 to 2010 including dengue cases, breeding percentage, temperature, humidity and population statistics are collated from the Minsitry of Health (MOH) (Weekly Infectious Diseases Bulletin; https://www.moh.gov.sg/content/moh_web/home/statistics/infectiousDiseasesStatistics/weekly_infectiousdiseasesbulletin.html), National Environment Agency (http://www.dengue.gov.sg/), and Department of Statistics, Singapore (2015) before being transformed and divided into sets that each contain predictors and a single output (dengue incidence at a future time point). In the third step, the LASSO method is employed to train and select the optimal models for future dengue forecast. In total, 12 models are generated, one per forecast week. In the final step, the 12 models are aggregated to make predictions over a 12-week window.

Covariates were considered at lags of up to 20 weeks based on the findings of a previous study by Hii et al. (2012a, 2012b), but in contrast to their approach, we allowed the effect of a single factor (such as temperature) to have multiple lags in influencing future dengue cases. The framework used in developing the models is presented in Figure 1.

### Other Approaches

We applied statistical methods used by Earnest et al. (2012) and Althouse et al. (2011) to compare with the LASSO approach described above.

In the Seasonal ARIMA (SARIMA) algorithm, models are composed of nonseasonal factors (*p, d, q*) and seasonal factors (*P, D, Q*), where *d* and *D* define the order (i.e., the number of weeks in the past) of nonseasonal and seasonal differencing in the time series (between successive values, used to reduce the effects of nonstationarity of the time series), *p* and *P* are the autoregressive (AR) terms, and *q* and *Q* are the moving average terms. SARIMA models can vary from very simple—for instance, a nonseasonal AR1 model in which the dependent variable is regressed upon itself (*y_t_* = β_0_ + β_1_ *y_t_*_– 1_ + ε*_t_*)—to very complex, where the dependent variable depends on several past weeks, on moving averages of past weeks’ data, and on recurrent seasonal factors, in the present case, 52 weeks ago. The values of (*p, d, q, P, D,* and *Q*) with the lowest Akaike information criterion (AIC) during model training are selected for the optimal model. [A definitive introduction is provided by Chatfield (2013).]

In the step-down linear regression (LR) model, we developed a submodel for each forecast window as in the LASSO approach, starting with a model containing all predictors (at multiple lags) and progressively eliminating variables one at a time according to AIC score, until no further improvement was possible without removing two or more terms simultaneously.

### Model Comparison

Models were compared using the mean absolute percentage error (MAPE), as proposed by Armstrong (1985) and modified and reproposed by Flores (1986), for each forecast window. If equation *D_t_*_+_ *_w_* is the actual number of dengue cases *w* weeks after time *t* when the prediction is made, and *D^m^_t + w_* is the number of cases forecasted by model *m*, the MAPE for that model and forecast window is


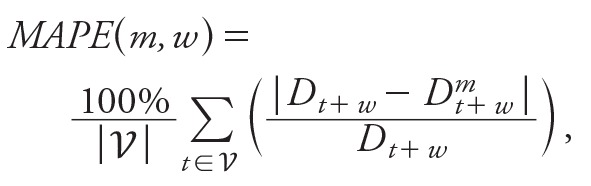
,[2]

where *V* is the validation set. We used data from 2001 to 2010 as training data to parameterize the models and from 2011 to 2012 to validate the models, and the validated models were applied to 2013 data to evaluate their performance. The same training and validation data sets were used for all three models.

Predictions were accompanied by 95% intervals using point estimates from the models with overlaid error using the estimated standard deviation of residuals from the fitted model, and we calculated the coverage of these intervals, that is, the proportion of time points in which the eventual data point fell within the prediction interval, to compare whether the actual coverage of the three approaches was at or above the nominal coverage.

### Data

Weekly covariates were used to match the frequency of reported dengue data made available by Singapore’s Ministry of Health, with finer resolution data coarsened to this level, and coarser data assumed to be homogeneous over each reporting interval. The time horizon used for all variables was January 2001 to December 2012 unless otherwise indicated. The variables used and their sources are described below:

***Case data.*** The weekly number of cases (natural log–transformed, +1) was provided by the Ministry of Health, Singapore, and can be obtained from their Weekly Infectious Diseases Bulletin (https://www.moh.gov.sg/content/moh_web/home/statistics/infectiousDiseasesStatistics/weekly_infectiousdiseasesbulletin.html).

***Population data.*** Midyear population sizes for residents and foreign non-residents were obtained for each year from the Singapore Department of Statistics (Department of Statistics, Singapore 2015), natural log–transformed, and applied to all weeks within the corresponding calendar year. These data were applied to all weeks within that calendar year.

***Meteorological data.*** Weekly mean temperature (*T*) in degrees Celsius, maximum hourly temperature, number of hours of high temperature (> 27.8°C) each week, and weekly relative humidity (RH) were obtained from Meteorological Services Singapore. Absolute humidity for any week (*H_A_*) was calculated from the weekly mean temperature (*T*) and the relative humidity (*H_R_*) using standard formulae (Xu et al. 2014).

***Vector surveillance data.*** The weekly breeding percentage (BP) is an in-house index developed by NEA that provides an estimate of the proportion of *Ae. aegypti,* the primary vector of dengue in Singapore, compared with all *Aedes* spp. As part of vector control operations, potential breeding sites are sought, samples are taken when breeding is found, and the species is determined in our laboratory. Because this is part of routine vector control and not solely for surveillance, efforts are not temporally or spatially regular, and they tend to favor outbreak periods and areas with transmission, thus biasing estimates upwards for both total incidence of breeding sites and *Ae. aegypti* breeding. To overcome the biases in data collection, we used the proportion of identified *Ae. aegypti* breeding out of all identified breeding sites to quantify the amount of “relevant” breeding. There are two justifications for this assumption: *a*) another *Aedes* species, *Ae. albopictus,* is so widespread that the amount of *Ae. albopictus* breeding found is a good proxy for total effort in identifying breeding sites, and *b*) *Ae. aegypti* is the primary vector for dengue in Singapore; this species is more efficient at transmission and more often found to be infected than *Ae. albopictus*, and the presence of *Ae. aegypti* is necessary for sustained transmission in any neighborhood. The breeding percentage for week *t*, *BP_t_*, was calculated from the weekly number of *Aedes* mosquito breeding sites recorded during ground inspections carried out by NEA using the following formula:


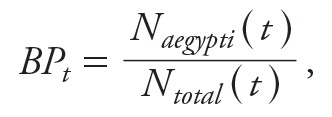
,[3]

where *N_aegypti_*(*t*) and *N_total_*(*t*) are the number of breeding sites containing only *Ae. aegypti* or containing either *Ae. aegypti* or *Ae*. *albopictus*, respectively, in week *t*. This index has been found to have a high correlation with dengue cases (unpublished data) and to be negatively correlated in space with chikungunya cases, for which *Ae. albopictus* is the more competent vector (Ng et al. 2009).

***Trend and seasonality data.*** In addition to climatic factors, dengue is affected by other factors such as changes to vector control and circulating serotypes. To address the impact of such nonclimatic factors on disease dynamics, we decomposed dengue incidence into terms for trend and for annual seasonality.

To account for changes, both gradual and abrupt, we extracted trends and seasonality from the weekly time series by using the Breaks For Additive Seasonal and Trend (BFAST) algorithm (Verbesselt et al. 2010). Specifically, BFAST decomposes time series into seasonality and trend components through iterative estimation of time series parameters and detection of break points, delimiting time windows in which different seasonal and trend patterns apply. Within each time window, the effect of seasonality is assumed to be sinusoidal, but the characteristics of the sinusoidal functions vary across time windows. Similarly, trend is defined to be piecewise linear between break points. The inferred seasonal and trend terms were extracted from BFAST and were allowed to be used as covariates in the LASSO model.

## Results

**Figure 2:**
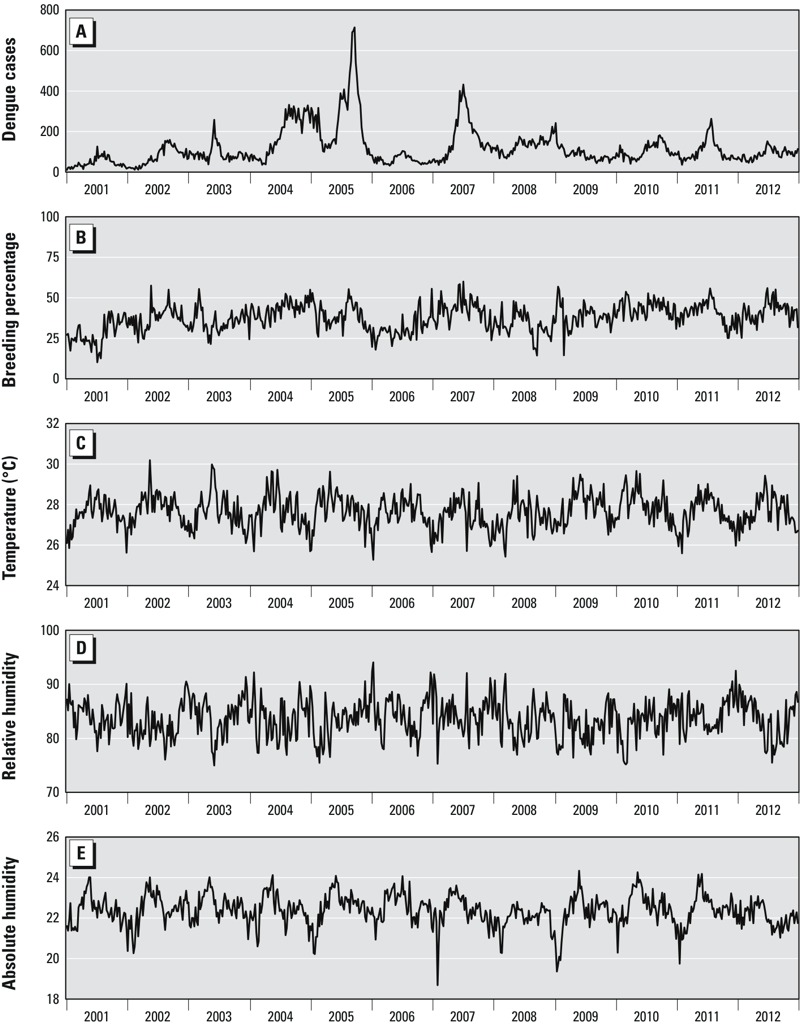
Model input from 2001 to 2012. (*A*) dengue incidence, (*B*) breeding percentage (BP), defined as the proportion of *Ae. aegypti*, the primary vector of dengue in Singapore, compared with all *Aedes* spp., (*C*) temperature, (*D*) relative humidity, and (*E*) absolute humidity from 2001 to 2012. Data sources are described in the text.

Data are presented in Figure 2. There was little overall variation in weather seasonality in Singapore over the time period investigated (2001 to 2010), with slightly hotter temperatures (around 1–2°C higher) and slightly higher absolute humidity registered in the middle of the year. Relative humidity did not display any notable patterns, and the breeding percentage (i.e., the relative amount of *Ae. aegypti*) varied without any clear pattern. In contrast, dengue fluctuated between low-level endemic and larger epidemic states.

**Figure 3:**
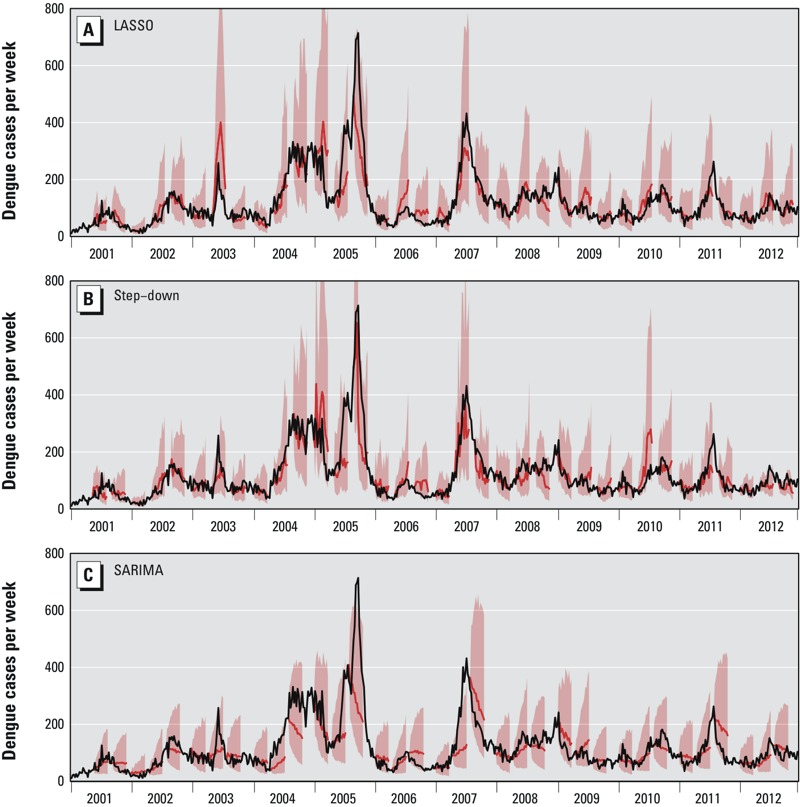
Comparison of dengue forecast from 2001 to 2012. Dengue forecast using (*A*) LASSO, (*B*) step-down linear regression and (*C*) SARIMA. For the LASSO, step-down linear regression and SARIMA methods, we selected all of the data (2001 to 2012) except the year being forecast to develop the model (to approximate the out-of-sample predictive performance in the other results). In each plot, the red lines represent model-based point estimates, and the pink contours represent the corresponding 95% prediction intervals. Each segment of predicted data (i.e., each pink and red region) represents the estimates from one 12-week forecast made at a previous point in time. Forecasts at other times are suppressed to simplify the figure but are presented in Video Files S1, S2, and S3.

In cross-validation, 12 sets of optimal model complexity parameters were selected for the 12 forecast windows. These included covariates whose effect was lagged from 1 to 20 weeks, and counting each lag separately in a total of 226 data streams, including seasonality and trend. We present the 12-week forecasts, including 95% prediction intervals, for various time points over the period 2001–2012 in Figure 3 for the LASSO method and the two other methods (step-down linear regression and SARIMA) (Dynamic 12-week forecasts for each model are presented in Video Files S1, S2, and S3). The LASSO and step-down approaches yielded forecasts that more accurately presaged short-term incidence than did the SARIMA model. The start and end of several epidemics were accurately forecasted by both the LASSO and step-down approaches, although the peak of the large 2005 outbreak was not well described by the LASSO model.

**Figure 4:**
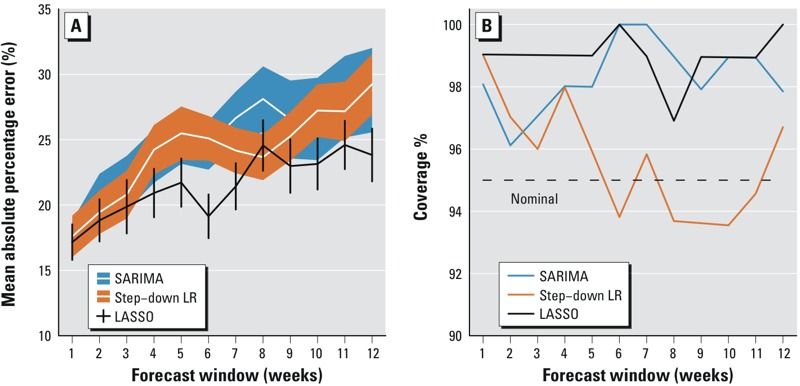
Comparison of model performance among SARIMA, step-down linear regression (LR) and LASSO using mean absolute percentage error (MAPE) and coverage of nominal 95% intervals. (*A*) MAPE comparison among LASSO, step-down linear regression and SARIMA over 1- to 12-week forecast windows. MAPE with 95% confidence intervals for LASSO, step-down linear regression and SARIMA are represented by vertical bars, filled orange polygons, and filled blue polygons, respectively. The MAPE degraded more slowly over time under the LASSO approach, with an increase from 17% error forecasting at 1 week to 24% error forecasting at 3 months, although the LASSO approach yielded comparable accuracy to those of step-down linear regression and SARIMA for the first 2 weeks. (*B*) Coverage of LASSO, step-down LR and SARIMA. For each forecast window period, the percentage coverage was calculated using the number of observations that fell within the 95% interval derived by overlaying the estimated error distribution on top of the forecast. The dashed line represents the nominal coverage of 95%.

The relative forecast accuracy was formally assessed by dividing the dataset into training (2001–2010) and validation sets (2011–2012) and comparing the MAPE of the best guess of the forecast and the coverage of the forecast interval. The results (Figure 4) support the use of LASSO to construct the forecasts: the LASSO approach yielded more accurate forecast time periods for all except the first 2-week window, in which the performance of the LASSO and step-down approaches were approximately equal. Notably, the MAPE degrades slowly over time under the LASSO approach, with a rise from 17% error [95% confidence interval (CI): 16, 19%] forecasting 1 week to 24% error (95% CI: 22, 26%) forecasting 3 months into the future. In contrast, both the step-down and SARIMA approaches had a MAPE of 29% at 3 months ahead (95% CI: 26, 32% [SARIMA], 27, 32% [step-down]). In addition, although the LASSO and SARIMA predictions were conservative in the sense that the actual coverage of prediction intervals exceeded the target of 95%, the step-down approach led to forecasts that understated the uncertainty, with a coverage that sometimes fell below the nominal level of 95% (Figure 4).

Interpretation of climatic and other factors was difficult because the strength of their association varied between forecast windows and because they operated over different time lags. However, recent dengue incidence (the autoregressive component) over a lag window of 1–5 weeks generally increased the forecast number of dengue cases; higher average weekly temperatures had a mostly dampening influence on forecast dengue cases, consistent with some findings in the literature (Morin et al. 2013); and the breeding percentage, reflecting the preponderance of *Ae. aegypti*, was mostly positively correlated with forecast dengue incidence. Dengue incidence over the next 4–5 weeks was positively associated with high levels of absolute humidity over the last month and negatively associated with high humidity 15–20 weeks previously.

**Figure 5:**
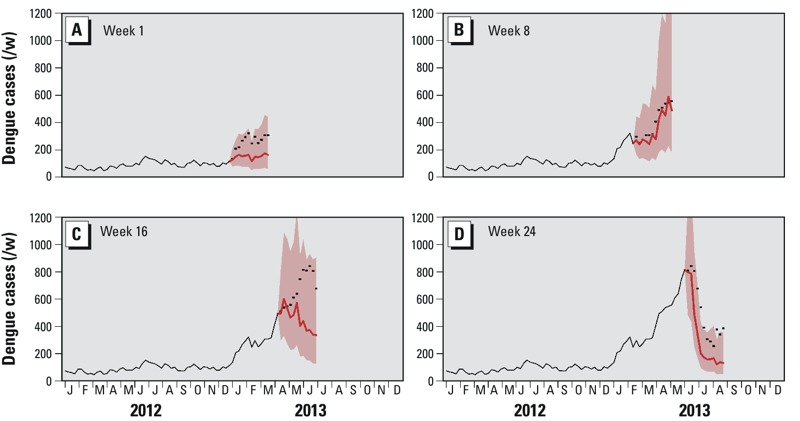
Illustration of model forecast in 2013 at weeks 1 (*A*), 8 (*B*), 16 (*C*) and 24 (*D*) using the LASSO method. In each panel, the *x*-axis represents time (2012/2013), and the *y*-axis represents the weekly number of dengue cases. The black lines indicate observed cases, the black dots indicate predicted (forecasted) cases, and the pink contours indicate the 95% intervals for the forecasts.

The forecasts at various time points in Singapore’s record-breaking 2013 epidemic, in which 22,170 cases were reported, are presented in Figure 5. Early in the epidemic (Figure 5A), the model forecast was of a mild rise, which was exceeded by the actual epidemic. By February (Figure 5B), the forecast was an almost perfect match to the data. At the end of April (Figure 5C), the forecast was of a decline, but the range of possible scenarios (the 95% interval for the forecast) included the subsequently observed peak at ~800 cases/week. The end of the epidemic, starting in July, was also successfully forecasted. Overall, the model predicted a slightly more rapid end to, and smaller size of, the epidemic than that which occurred.

## Discussion

Penalized regression, of which LASSO is the most prominent methodology, is an idea that dates back to the 1990s (Tibshirani 2011) and over the last decade has led to substantial theoretical and methodological advances in “small *n* large *p*” problems in which the number of observations is smaller than the number of potential predictors. By optimizing a combination of model goodness of fit (via the likelihood) and model complexity (via a penalty that grows with the size of the parameters in the model), and using cross-validation to identify the optimal penalty term, penalized regression can simultaneously handle both model building and parameter estimation, avoid over-fitting, and improve out-of-sample predictive accuracy (Hoerl and Kennard 1970; Zhao and Yu 2006). By shrinking regression coefficients of less importance to or towards zero and thereby retaining only the most important predictors, LASSO is able to obtain good interpretability and stability (Zhao and Yu 2006).

Although LASSO is a well-established methodology that is frequently used in bioinformatics and other big-data applications (González-Recio et al. 2009; Shi et al. 2007; Wu et al. 2009), there are few applications of this method in epidemiology (for instance, Walter and Tiemeier 2009) or in neglected tropical diseases. In this paper, we report the use of LASSO in forecasting an endemic and high-burden disease—dengue—in Singapore, which, by virtue of its location near the equator and concomitant lack of seasonality, experiences unpredictable outbreaks above the usual endemic level. The LASSO methodology has several advantages over traditional approaches: *a*) Model selection is rapid (approximately 2 min), automatic and objective, in contrast to more laborious and sometimes subjective approaches such as the Hosmer and Lemeshow (2000) purposeful approach to model building, or common approaches such as forward selection using *p*-values (Grechanovsky and Pinsker 1995). *b*) Tautologically, by selecting the model complexity using cross-validation to optimize predictive performance, predictive performance of the routine is optimized; thus, the task of making better and more accurate forecasts is reduced to that of finding potentially informative covariates. *c*) LASSO allows large numbers of possible predictors to be considered without prejudicing the accuracy of the routine because nonpredictive variables obtain zero coefficients for optimal values of the penalty term and thereby drop out of the final model. *d*) By using distinct models for 1-week forecasts, 2-week forecasts, and so on, the variables used and the values of their associated coefficients can be tailored to the specific requirements of forecasting different lengths of time into the future. Recent dengue cases (over the last 4 weeks) are important in forecasting for 1–12 weeks, and average temperature is not useful in short-term forecasts (1–3 weeks), although it is for longer forecasts (4–12 weeks); these examples highlight the need for separate submodels for different forecast windows. Having distinct submodels also obviates the need to forecast future values of the predictors, which would be the case if a single model for 1 week ahead were used and then iterated to obtain longer-term forecasts. This approach led to high accuracy for both immediate (next week, MAPE 17%) and long-term (3 months, MAPE 24%) predictions. Although the forecast accuracy degraded as the forecast window was extended (see Figure 4), this degradation was surprisingly slight, and we were able to predict the large outbreaks of both 2013 and 2014 over 10 weeks in advance, giving advance warning to allow operations to be rolled out. We restricted the forecast window to 12 weeks to avoid the increased level of inaccuracy that accompanies long-term projection and because short- (several weeks) and medium-term (several months) projections are the most useful for local planning purposes.

There are, however, some limitations to this approach. The largest of these limitations is that although very good predictive accuracy can be achieved, the 12 models built using the LASSO method are not amenable to interpretation because they were constructed for their predictive accuracy, not to explain the etiology of outbreaks. In particular, attempts to explain to stakeholders why the model forecast a large epidemic in 2013 were hindered by the numerous covariates acting at different lags. Interpretation is increasingly difficult at longer forecast windows. For example, ~60 predictors out of the complete set of > 200 were selected for the 12-week-ahead submodel. Among these 60 variables, the same covariate was often selected at different lags and frequently was selected with differently signed coefficients at those different lags. The complexity needed for good forecasts reflects the multitude of factors operating on the vector and virus–vector interactions. One plausible way to reduce the apparent complexity would be to combine our approach with a mechanistic model of drivers of the mosquito life cycle, for instance, via the Focks model (Focks et al. 1995), with output from the mechanistic model replacing some or all of the environmental drivers in the statistical model. The variables we used include meteorological data, case data, vector surveillance data, and human population data. Other relevant indicators of risk, particularly on circulating serotypes, genotypes, and evidence on herd immunity via occasional sero-epidemiological surveys, may subsequently be incorporated. However, because comprehensive analysis of genotype and serotype of dengue cases in Singapore began in approximately 2006, and because testing protocols have evolved since that time, we need to explore the best way to incorporate these sources of information.

The forecasting tool described in this paper has become an integral part of Singapore’s dengue control program. The final model is embedded in a “real-time” schedule, with data (at present) being updated weekly and predictions sent out to our operational partners (examples of the forecasts used in the 2013 outbreak are shown in Figure 5), including the Ministry of Health and the Environmental Public Health Operations Department of the NEA. During the 2013 epidemic, our forecasts helped guide hospital bed management and public health interventions, including preemptive source reduction measures, recruitment of ground staff, and education campaigns. In late March 2013, our models forecasted an earlier-than-usual increase in dengue cases in June 2013, which could potentially peak at 800 cases/week. Specifically, the forecast predicted a peak in case count of 863 during the 26th week of 2013, which is very close to the observed number of cases, which peaked at 842 cases/week during the 25th week. In addition to aiding with resource planning, this forecast also facilitated early risk communication to the public and the advanced launch of Dengue Campaign in April, 2 months ahead of its traditional June launch.

## Conclusion

Future work will automate the data-handling process so that predictions can be generated and posted online without the routine being rerun manually; such automation will also allow the forecast to be made daily and hence to be genuinely in “real time.” Extending the forecasts beyond 12 weeks may be challenging because some of the key drivers, such as local weather conditions, may have a short-term but strong effect on dengue that requires integrating the predictive model with weather forecasting models, where long-term forecasts may not be readily available.

## Supplemental Material

(3.3 MB) ZIP
