# Supplementary material for: Three-Month Real-Time Dengue Forecast Models: An Early Warning System for Outbreak Alerts and Policy Decision Support in Singapore
Source: Environ Health Perspect. 2015 Dec 11;124(9):1369–75. doi: 10.1289/ehp.1509981 (PMC5010413; doi:10.1289/ehp.1509981)
Supplement: (3.3 MB) ZIP [file ehp.1509981.s001.acco.zip › Supplemental Video Index File.pdf]

Environ Health Perspect
DOI: 10.1289/ehp.1509981

Three-Month Real-Time Dengue Forecast Models: An Early Warning System for Outbreak Alerts and Policy Decision Support in Singapore

Yuan Shi, Xu Liu, Suet-Yheng Kok, Jayanthi Rajarethinam, Shaohong Liang, Grace Yap, Chee-Seng Chong, Kim-Sung Lee, Sharon S.Y. Tan, Christopher Kuan Yew Chin, Andrew Lo, Waiming Kong, Lee Ching Ng, and Alex R. Cook

Supplemental Video Index

Video File S1*: Comparison between observed cases and predicted cases from 2001-2012 using LASSO. In the video, we selected all the data (2001 to 2012) except the year being forecast to develop the models. The results present the 12-week forecasts, including 95% prediction intervals, at various time points over the period. The same strategy also applies to the videos generated by step-down and SARIMA methods (video S2 and S3).

Video File S2*: Comparison between observed cases and predicted cases from 2001-2012 using step-down linear regression

Video File S3*: Comparison between observed cases and predicted cases from 2001-2012 using SARIMA

*In all the three videos, black lines represent past cases, and red circles represent future cases. Red lines represent model-based point estimates and the pink contours represent corresponding 95% prediction intervals. Each segment of predicted data (i.e., each pink and red region) represents the estimates from one 12-week forecast made at a previous point in time.
